# L2S2: chemical perturbation and CRISPR KO LINCS L1000 signature search engine

**DOI:** 10.1093/nar/gkaf373

**Published:** 2025-05-01

**Authors:** Giacomo B Marino, John E Evangelista, Daniel J B Clarke, Avi Ma’ayan

**Affiliations:** Department of Pharmacological Sciences, Department of Artificial Intelligence and Human Health, Mount Sinai Center for Bioinformatics, Icahn School of Medicine at Mount Sinai, New York, NY 10029, United States; Department of Pharmacological Sciences, Department of Artificial Intelligence and Human Health, Mount Sinai Center for Bioinformatics, Icahn School of Medicine at Mount Sinai, New York, NY 10029, United States; Department of Pharmacological Sciences, Department of Artificial Intelligence and Human Health, Mount Sinai Center for Bioinformatics, Icahn School of Medicine at Mount Sinai, New York, NY 10029, United States; Department of Pharmacological Sciences, Department of Artificial Intelligence and Human Health, Mount Sinai Center for Bioinformatics, Icahn School of Medicine at Mount Sinai, New York, NY 10029, United States

## Abstract

As part of the Library of Integrated Network-Based Cellular Signatures (LINCS) NIH initiative, 248 human cell lines were profiled with the L1000 assay to measure the effect of 33 621 small molecules and 7508 single-gene CRISPR knockouts. From this massive dataset, we computed 1.678 million sets of up- and down-regulated genes. These gene sets are served for search by the LINCS L1000 Signature Search (L2S2) web server application. With L2S2, users can identify small molecules and single gene CRISPR KOs that produce gene expression profiles similar or opposite to their submitted single or up/down gene sets. L2S2 also includes a consensus search feature that ranks perturbations across all cellular contexts, time points, and concentrations. To demonstrate the utility of L2S2, we crossed the L2S2 gene sets with gene sets collected for the RummaGEO resource. The analysis identified clusters of differentially expressed genes that match drug classes, tissues, and diseases, pointing to many opportunities for drug repurposing and drug discovery. Overall, the L2S2 web server application can be used to further the development of personalized therapeutics while expanding our understanding of complex human diseases. The L2S2 web server application is available at https://l2s2.maayanlab.cloud.

## Graphical Abstract

**Graphical Abstract:**
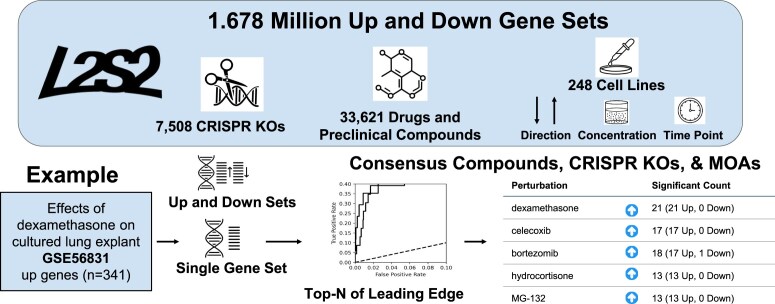

## Introduction

Gene expression profiling of cells before and after treatment with a large collection of chemical and genetic perturbations is a powerful approach for establishing a connectivity mapping resource that can be used for drug discovery, drug repurposing, and adverse event predictions [1]. With the introduction of genome-wide microarrays, a connectivity mapping resource was first created using yeast [2]. This resource provided data collected by treating *Saccharomyces cerevisiae* with ∼300 mutational and chemical perturbations. Several years later, a similar approach was employed to create a more expansive resource named Connectivity Map (CMAP) [3]. To create CMAP, four human cancer cell lines were treated with ∼1000 drugs, mostly FDA approved, in different concentrations, and then gene expression data was collected with microarrays after six hours of drugs treatment. The resource became widely popular partially due to a web server search engine application that was developed to provide access to the data. Users of this web server application were able to input their gene expression signatures to receive matched signatures that may reverse or mimic their input expression vectors. Following the success of CMAP, the Library of Integrated Network-based Cellular Signatures (LINCS) NIH Common Fund program was established [4]. The LINCS program systematically profiled the response of human cells to drugs, small molecules, gene knockouts, gene knockdowns, gene over-expressions, micro-environments, and disease perturbations followed by multi-omics assays such as transcriptomics, epigenomics, and proteomics, as well as cellular microscopy imaging. The LINCS data were made available for download, browse, and search via several data portals developed by members of the LINCS consortium [5–7].

One of the most expansive datasets produced by the LINCS program is the profiling of 248 human cell lines measuring gene expression changes in response to 33 621 small molecules and 7508 clustered regularly interspaced short palindromic repeats (CRISPR) knockouts (KOs) of single genes using the L1000 assay [8]. Several search engines were developed specifically for LINCS L1000 data. These search engines have advantages and disadvantages and were developed over several years supporting the evolving collection of subsets of the L1000 dataset. The producers of the L1000 data, the CMAP team at the Broad Institute, developed the clue.io platform. The clue.io platform includes a collection of datasets and tools that can be used to analyze, explore, and visualize the L1000 data all in one place [4]. The LINCS Canvas Browser provided visualizations of clustered LINCS L1000 signatures with matching enrichment analysis results [9]. The L1000 Viewer enabled users to search the L1000 signatures by specific attributes such as cell line, concentration, and time point [10]. iLINCS [11] offers interactive analysis and visualization tools for over 200 000 precomputed signatures, including LINCS L1000 data. L1000CD^2^ [12] supports up- and down-gene set searches to identify mimickers and reversers using signatures computed with the characteristic direction (CD) method [13]. L1000FWD [14] provides an interactive two-dimensional map of ∼17 000 L1000 signatures, sortable by attributes such as cell line, mechanism of action (MoA), and clinical phase. Both L1000CD^2^ and L1000FWD serve for search only a subset of the L1000 data filtered to only include the most consistent and impactful signatures. For L1000FWD, an extra data normalization step was added. SigCom LINCS [15] is another L1000 signature search engine that serves all the available and most recent L1000 signatures. SigCom LINCS also serves automatically computed signatures from the Gene Expression Omnibus (GEO) [16] and GTEx [17]. The signatureSearch R package provides local access to enrichment analysis and correlation-based gene expression signature search, as well as corresponding visualizations, of the LINCS L1000 and the original CMAP datasets [18]. More recently, the DOSE-L1000 Visualizer was published, facilitating volcano plot visualizations of compounds, genes, dose–response curves, and efficacy versus potency plots [19]. Moreover, the Bioconductor R package Slinky was developed to analyze and visualize the LINCS L1000 data via a command line interface [20]. We summarize the features and average query time for the tools mentioned above and other related resources that provide L1000 signature and gene set search (Table 1).

**Table 1.** Features and attributes of publicly available L1000 search engines and related software packages. * denotes deprecated URL

Resource	URL	Available Online	Signature Search	Consensus Perturbations	Interactive Visualization of Results	Query Time
**L2S2**	https://l2s2.maayanlab.cloud/	✓	✓	✓	✗	150 genes versus 1.678M gene sets, < 1s 300 up/down genes versus 1.678M signatures, ∼1 s;
**SigCom LINCS**	https://maayanlab.cloud/sigcom-lincs/	✓	✓	✓	✓	150 genes versus ∼1.5M signatures, ∼10 s 300 up/down genes versus ∼1.5M signatures, ∼25 s;
**Clue**	https://clue.io/	✓	✓	✗	✓	150 genes versus ∼420k signatures, ∼30 min 300 up/down genes versus ∼420k signatures, ∼30 min
**iLINCS**	https://www.ilincs.org/ilincs/	✓	✗	✗		150 genes versus 227k signatures, ∼40 s 300 up/down genes versus 227k signatures, ∼25 s
**L1000CD2**	https://maayanlab.cloud/L1000CDS2/	✓	✓	✗	✗	300 up/down genes versus 20k signatures, <1 s
**L1000FWD**	https://maayanlab.cloud/l1000fwd/	✓	✓	✗	✓	300 up/down genes versus 20k signatures, ∼20 s
**LINCS Canvas Browser**	http://www.maayanlab.net/LINCS/LCB/ *	✗	✓	✗		N/A
**L1000 Viewer**	http://l1000viewer.bio-complexity.com/*	✗	✗	✗		N/A
**Signature Search**	https://bioconductor.org/packages/release/bioc/html/signatureSearch.html	✗	✓	✗	✓	N/A
**Slinky**	https://bioconductor.riken.jp/packages/3.8/bioc/html/slinky.html	✗	✗	✗	✗	N/A
**DOSE-L1000**	https://dosel1000.com/	✓	✗	✗	✓	N/A

Here, we present the web server application LINCS L1000 Signature Search (L2S2), a platform that enables rapid gene set searches across 1.678 million up- and down-gene sets derived from LINCS L1000 CRISPR KO and chemical perturbation data. Given the vast number of signatures associated with each perturbation, L2S2 also provides consensus perturbation search. To explore the pharmacological space of the gene sets served by L2S2, we queried a large randomly sampled subset of the gene sets served by RummaGEO [21] with L2S2 to identify single drugs and drug classes that are highly ranked in different RummaGEO gene set clusters. Such analysis uncovered relationships between drugs and preclinical small molecules, with tissues, diseases, and MoAs.

## Materials and methods

### The L2S2 search engine

To receive near-instant results when searching through 1.678 million gene sets, a super-fast enrichment analysis search engine is implemented in Rust. The search engine is utilizing bit-vectors stored in random access memory (RAM) for fast computation of the Fisher’s exact test given the query gene set, and all the 1.678 million L2S2 gene sets. This enrichment analysis search engine was previously described [22]. For the L2S2 implementation, the Rust API was modified to enable up/down gene set queries. User query gene set pairs are submitted to the Rust API to be compared to each gene set pair in the L2S2 database. First, a mimicker and a reverser overlap is computed for each pair. A mimicker is defined as the overlap between the user-submitted up-gene-set and the L2S2 up-gene-set added to the overlap between the user-submitted down-gene-set and the L2S2 down-gene-set. A reverser is similarly the addition of the overlapping genes from the user-submitted up-gene-set and the L2S2 down-gene-set, and the overlapping genes from the user-submitted down-gene-set and the L1000 up-gene-set. These overlaps fill the entries in the contingency table for computing the Fisher’s exact test, along with the size of the user’s query up and down sets, the total size of the L2S2 up- and down- gene-sets, and the background (Supplementary Fig. S1A). Benjamini–Hochberg (BH) adjusted *P-*values are additionally computed and reported to address the concern of multiple hypotheses testing.

### L2S2 consensus search

Consensus perturbations and consensus MoAs are computed for each user-submitted gene set or up/down gene set pair. For single gene sets, the Fisher’s exact test is used to compare the number of significant up signatures (in the top-N signatures, with the default set to *n* = 10 000), the number of significant down signatures, the number of insignificant up signatures, and the number of insignificant down signatures, as *a*, *b*, *c*, and *d* in the contingency table respectively (Supplementary Fig. S1B). To compute consensus down signatures, the entries are flipped accordingly. For single user-submitted gene sets, each up and down consensus perturbation are tested independently. A similar procedure is employed for user-submitted gene set pairs wherein significant “mimicker” and “reverser” signatures are counted in the top-N, instead of the “up” or “down” signatures. BH adjusted *P*-values are computed and reported for each consensus perturbation to correct for multiple hypotheses testing.

### Benchmarking different consensus perturbation ranking methods

To benchmark various strategies to compute consensus perturbations, up- and down- gene-sets were first extracted from the gene expression and enrichment vector analyzer (GEN3VA) [23] resource. GEN3VA contains manually curated microarray gene expression signatures sourced from GEO [16] utilizing the tool GEO2Enrichr [24]. Differential gene expressions following drug, gene, and disease perturbations in GEN3VA were computed using the CD method [13]. From the collection of GEN3VA signatures, we extracted up- and down- gene-sets for three perturbations: dexamethasone (*n* = 86), thiazolidinedione (*n* = 94), and tamoxifen (*n* = 66). We next assessed the ability of L2S2 to recover these compounds by ranking L1000 consensus perturbations using different methods. The different methods rank up- and down- gene-sets individually, as well as up/down pairs. The ranks of the recovered perturbations are compared with receiver operating characteristic (ROC) curves, and the area under the curves (AUCs).

As described above, we computed the proportion of the number of perturbations in the top-N ranked perturbations compared to the number of the same perturbations in the remaining signatures. By changing the size of *N*, we evaluated how the size of the leading edge of the enrichment results relates to the quality of the consensus ranking ability to recover the given perturbation. The AUCs for the consensus perturbations were compared to three other methods for that rank consensus L1000 perturbations for single and up/down gene set queries. Using the results from SigCom LINCS [15], we implemented the Kolmogorov–Smirnov (KS) test to compare the ranks of specific compounds, or specific genetic perturbations, to both uniform and normal distributions. The two-tailed KS test statistic was then used to rank the consensus perturbation. Similarly, the Mann–Whitney (MW) U test was employed to the full ranks of enriched perturbations from SigCom LINCS to rank consensus signatures based on each consensus perturbation MW U rank sum statistic. Because all three methods require multiple entries for each perturbation to be significant, only perturbations with at least 10 signatures are included in the consensus rankings. In addition, since the API only returns significant perturbations with a threshold of *P*< 0.05, some ROC curves have fewer entries for the expected drug.

### UMAP visualization of the RummaGEO signatures

The human gene sets from RummaGEO [21] were systematically submitted to the L2S2 API. Each RummaGEO gene set was then associated with the top 10 enriched perturbations from L2S2. Utilizing scikit-learn [25], each submitted gene set was transformed into a one-hot encoded vector. Truncated singular value decomposition (SVD) [26] was applied to reduce the dimensionality of the inverse document frequency (IDF) vectors to 50 singular values. Next, UMAP [27] was used with default parameters to project these vectors onto a two-dimensional space. MoAs for compounds were sourced from Drug Repurposing Hub [28]. Clustering for functional analysis was performed with the Leiden algorithm [29] (n_neighbors = 30, resolution = 0.5), and the overlapping genes of each top-ranked enriched perturbation were aggregated. Genes appearing in a significant amount of overlaps, defined as those genes with counts two standard deviations from the mean, were retained and submitted to Enrichr for functional elucidation [30] (Supplementary Table S1). To further annotate the Leiden identified clusters with disease and tissue terms, the large language model (LLM) Mistral-7B-Instruct-v0.2 [31] was used to extract keywords from PubMed abstracts associated with the GEO studies in each cluster. These key terms were previously extracted for RummaGEO [21] to enable term enrichment analysis. The disease and tissue mentions were then summed to compute a percentage of studies in each cluster that mentioned a disease or a tissue term. The thresholds 10% for MoAs and 5% for diseases were applied to only highlight clusters with commonly appearing terms.

### Implementing the L2S2 web server application

L2S2 is implemented using Typescript and Tailwind CSS in the NextJS framework. It is connected to a PostgreSQL database, which is queried through a GraphQL endpoint enabled by Postgrapile and Apollo for type-safe queries. Enrichment results are retrieved from the database using the Rust API described in the search engine implementation.

## Results

### The L2S2 web server application

**Figure 1.**
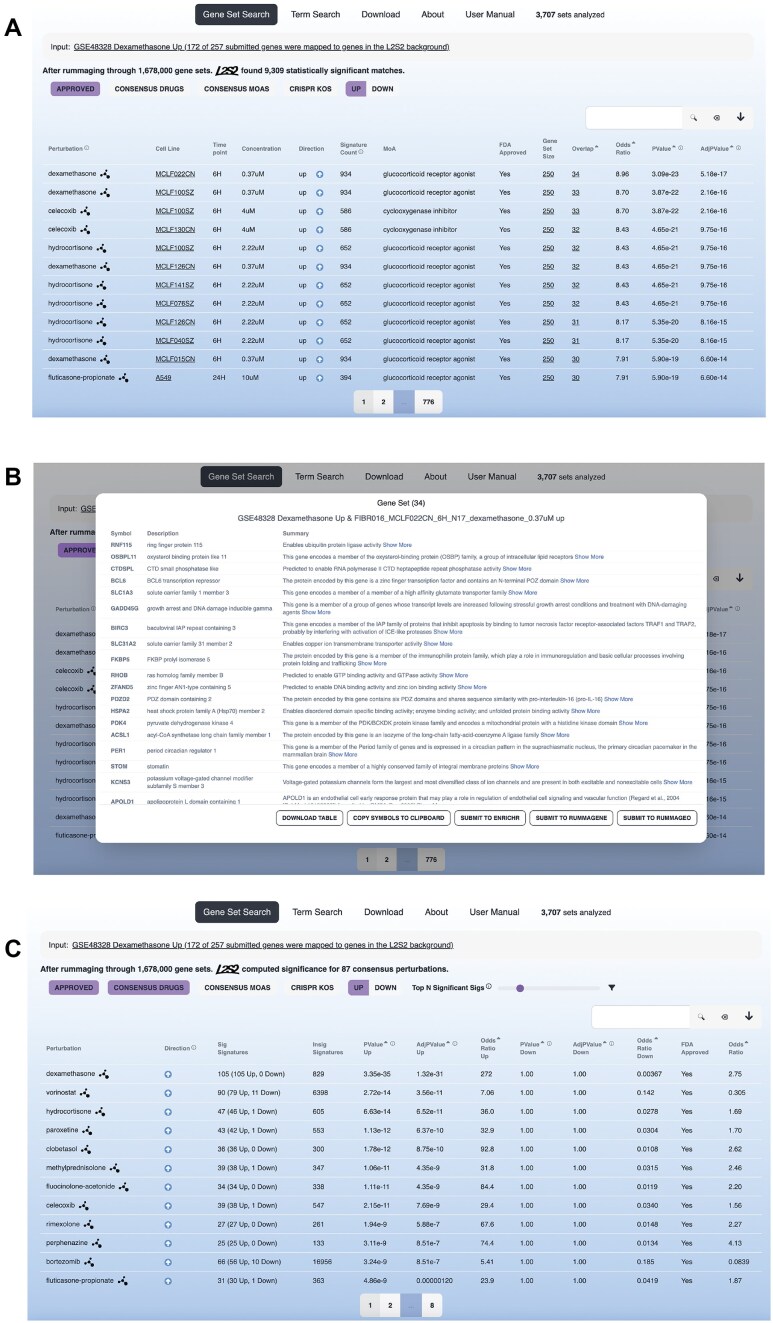
The L2S2 results page after submitting a query of up-regulated genes (*n* = 257) from a GEO study (GSE48328) that profiled the HEK293 cell lines with either a wild-type glucocorticoid receptor (GR) or a SUMOylation-defective GR. (**A**) Enrichment results from the Fisher’s exact test with filters for up gene sets and FDA-approved drugs. (**B**) Overlapping genes between the input gene set and the top returned perturbagen dexamethasone in the MCLF022CN cell line. (**C**) Consensus enrichment results for perturbations across cell lines and time points.

L2S2 is an enrichment analysis connectivity mapping search engine that queries 1.678 million chemical perturbations and CRISPR KO L1000 LINCS up and down gene sets. Through a user-friendly interface, users can submit either a single gene set, or a pair of up/down sets, to identify significantly matching gene sets as potential reversers or mimickers. Results are displayed in a paginated table, including details about the perturbation, cell line, time point, concentration, directionality, number of signatures for that perturbation in the database, the gene set associated with the perturbation, number of overlapping genes, a list of the overlapping genes, and enrichment statistics (Fig. 1A). Users can filter the results by FDA-approved drugs, CRISPR KOs, signature directionality, or any search term, for example, the name of a drug. To assist users with interpreting the results, compounds are linked to PubChem [32], and genes from CRISPR KOs are linked to PrismEXP [33] and Harmonizome [34, 35]. Both Harmonizome and PrismEXP provide functional knowledge about genes, while PrismEXP also has function predictions based on co-expression correlations. Clicking on the gene set size, or the overlap size, in the results table, opens a modal window where users can inspect each gene set in more detail, including viewing gene description summaries from NCBI, downloading the gene set, and copying the gene symbols to the clipboard. In addition, the modal window also includes buttons to submit the identified genes to Enrichr [30], which hosts thousands of annotated gene sets; Rummagene [22], which hosts automatically extracted gene sets from PubMed Central (PMC) supplementary material tables; and RummaGEO [21], which provides automatically computed gene expression signatures from GEO (Fig. 1B). Users can also explore the consensus perturbation results, and adjust the number of significant signatures included in the computation of the consensus signatures by moving a slider above the table (Fig. 1C). All the results tables can be downloaded by clicking on the icon next to the search bar above the results table. Additionally, L2S2 offers term search to locate relevant signatures within the results table. L2S2 also has a download page, a user manual with step-by-step guide, and detailed documentation on accessing L2S2 via the GraphQL API.

### Benchmarking methods to compute consensus perturbations

**Figure 2.**
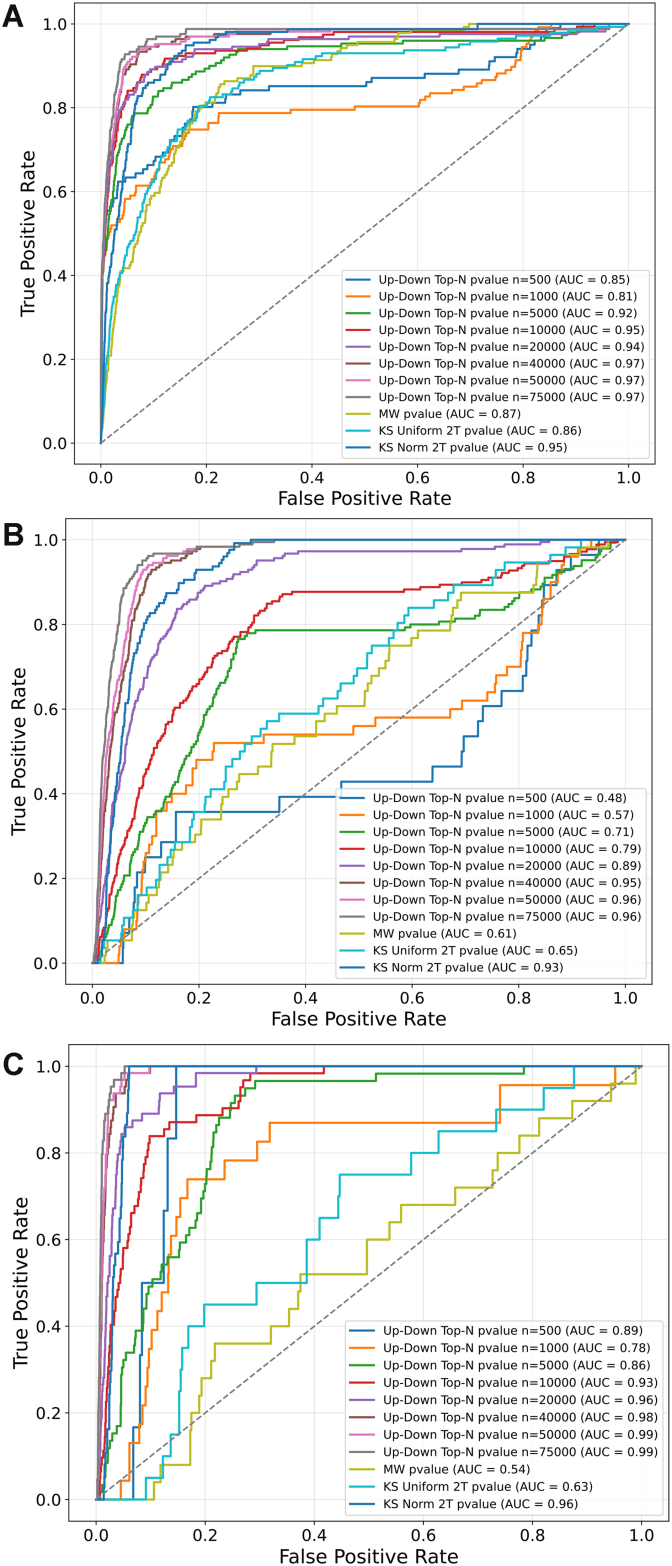
Benchmarking different methods to compute consensus signatures. The Top-N consensus method with varied window size (*N* = 500–75 000) is compared to consensus methods that use the KS test with either uniform or normal distributions, and the MW test. ROC curves of L2S2 ranking of a specific drug when querying L2S2 with up/down gene sets from manually curated signatures created from GEO microarray studies that used the same drug in various contexts. (**A**) dexamethasone, GEN3VA (*n* = 86); (**B**) thiazolidinedione, GEN3VA (*n* = 94); and (**C**) tamoxifen, GEN3VA (*n* = 66).

Since L2S2 commonly returns thousands of significant results, it is often difficult to assess whether a top ranked drug, preclinical small molecule, or a genetic perturbation would succeed in inducing a desired effect in a user experimental setting in a different cellular context. This is especially relevant if the same perturbation in a different L1000 context, i.e., concentration, cell line, and time point, is not highly ranked or significant for the same query. To address this concern, L2S2 offers consensus signature search. Consensus perturbation rankings aggregates the results across all the 1.678 million gene sets served by L2S2. To benchmark various consensus signature ranking approaches, we used a subset of up/down gene sets from GEN3VA [23]. GEN3VA contains manually curated gene expression signatures for drug perturbation extracted from microarray studies in GEO. Specifically, signatures for three drugs are used: dexamethasone (*n* = 86), thiazolidinedione (*n* = 94), and tamoxifen (*n* = 66). Each up/down gene set pair from GEN3VA was submitted to L2S2, and then the ranks of the given drug were compared by plotting ROC curves. The Top-N method was compared against the KS test using uniform and normal distributions, and the MW test to recover the given drug. The highest AUCs achieved across all three drugs are from the Top-N consensus method with *N* = 50 000 (Fig. 2). In each case, the Top-N method outperformed the MW and KS tests, although the KS test utilizing the normal distribution for comparison performed highly in all three cases (AUC ≥ 0.93).

**Figure 3.**
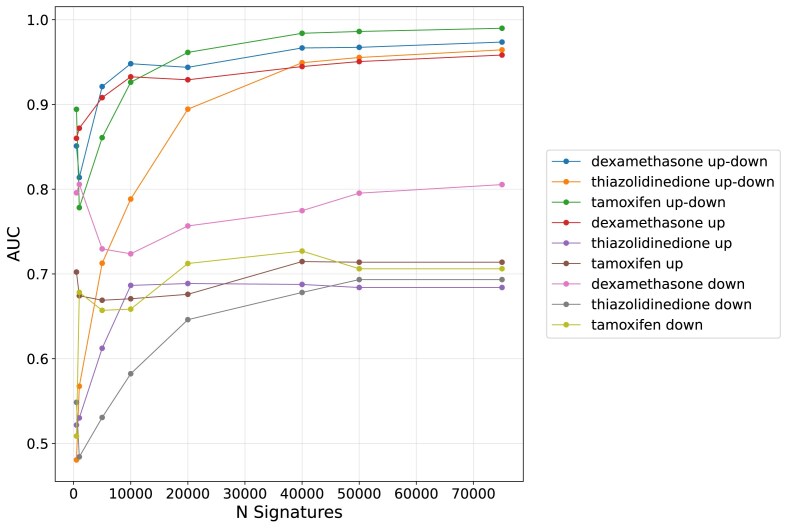
AUCs as a function of the Top-N signatures used for consensus perturbation *P*-value computation measured at *N* = 1000, 5000, 10 000, 20 000, 40 000, 50 000, and 75 000. Each benchmarking set from GEN3VA, dexamethasone (*n* = 86), thiazolidinedione (*n* = 94), and tamoxifen (*n* = 66) up and down gene sets were submitted separately and in pairs.

Additionally, up and down gene sets were submitted separately to L2S2 to assess the accuracy of the consensus results for single-gene set searches (Supplementary Fig. S2). In this case, up-regulated genes following dexamethasone exposure perform best (AUC = 0.96, *N* = 75 000) (Supplementary Fig. S2A), while for both thiazolidinedione and tamoxifen, lower AUCs are observed. Many of the ROC curves exhibit a bimodal distribution. This indicates that a matching profile is better captured by an up/down gene sets query. This is likely due to the drug's MoAs and impact on its cellular targets. Dexamethasone is known to directly activate the glucocorticoid receptor (GR) which then acts as a mostly activating transcription factor of its targets [36, 37]. In contrast, thiazolidinedione, an anti-diabetes drug used to increase insulin sensitivity, is known to have a more complex MoA and multiple targets. Thiazolidinedione regulates gene expression mainly through peroxisome proliferator-activated receptor gamma (PPAR-γ) activation but also has PPAR-γ independent effects, for example, on skeletal muscle fuel metabolism [38, 39]. Thiazolidinedione is also a partial GR agonist, exhibiting similar but less potent effects than dexamethasone [40]. Tamoxifen is a selective estrogen receptor (ER) modulator used to treat ER-positive breast cancer [41]. Tamoxifen’s effects on gene expression are less understood but it is known that this drug’s MoAs involve multiple pathways, and it takes longer for tamoxifen to produce significant changes. Given the increased recovery of the expected drugs with increased significant *N* signature windows, we plotted the AUCs as a function of *N* (Fig. 3). We observe that although a small *N* can produce high AUC for some drugs, AUCs largely increase until a window size of 20 000–30 000, and then the performance flattens. This analysis also shows that the up/down paired sets achieve better recovery of the target compound, except for the dexamethasone up sets, which performs similarly to the up/down set pairs.

#### Use case 1: I*dentifying consensus compounds that reverse the expression of genes up-regulated following CYTOR knockdown in the K562 cell line*

**Figure 4.**
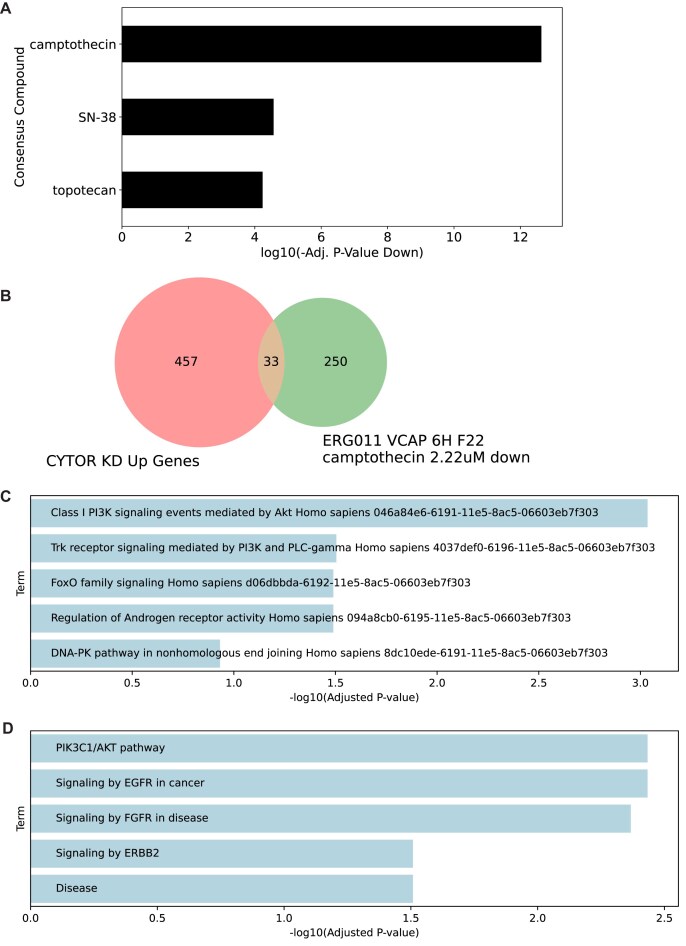
Identifying consensus compounds that reverse the expression of genes up-regulated following CYTOR knockdown in K562 cell lines (GSE285084). (**A**) L2S2 consensus compounds ranked by adjusted *P*-value for down-regulated signatures CYTOR KD Up (*n* = 457 adj. *P*-value < 0.01; logFC > 0). (**B**) Enrichment results for the overlapping genes (*n* = 33) between CYTOR KD Up (*n* = 500) & RAD001_HCC515_6H_H14_camptothecin_10 μM down (*n* = 250) from the NCI-Nature 2016 library from Enrichr. (**C**) Enrichment results for the overlapping genes (*n* = 33) between CYTOR KD Up (*n* = 500) & RAD001_HCC515_6H_H14_camptothecin_10 μM down (*n* = 250) from the BioPlanet 2019 library from Enrichr.

Long noncoding RNAs (lncRNAs) are implicated in multiple cancers and are often linked with chemoresistance and poor prognosis. One of the most highly studied lncRNA is the cytoskeleton regulator RNA (CYTOR). CYTOR is known to be up-regulated in many different cancers, including gastric, lung adenocarcinoma, renal cell carcinoma, and other cancers, and its expression is significantly associated with poor survival as well as lymph node metastasis [42]. Since CYTOR is known to play a role in chemo-resistance after radiotherapy, gene expression was profiled in K562 cell lines following CYTOR knockdown with CRISPR interference (CRISPRi) (GSE285084) [43]. To better understand the mechanistic action of CYTOR and potentially identify small molecules and drug targets that can mitigate its chemo-resistance effects, we utilized the L2S2 platform to identify reversers and mimickers of the observed expression following its knockdown in K562 cells. First, we identified the up-regulated genes following the CYTOR knockdown (BH adjusted *P*-value < 0.01, logFC > 0.5) (*n* = 457) and submitted these genes as a query to L2S2. The most highly ranked drug from this query is camptothecin (Fig. 4A), an anticancer drug that is known to inhibit topoisomerase I, an enzyme crucial for DNA repair [44]. Campthoecin has shown antitumor effects in multiple cancer models, including colorectal cancer, non-small-cell lung cancer, small-cell lung cancer, pancreatic cancer, and breast cancer [45]. To further investigate the shared mechanism of camptothecin and CYTOR KD, we analyzed the function of the overlapping genes (*n* = 33) between the up-regulated genes following CYTOR knockdown and the most significant individual camptothecin signature (VCAP cell line, 6H time point, 2.22 μM, adjusted *P*-value 6.44 × 10^–8^) (Fig. 4B). Submitting these genes to Enrichr [30] reveals significant enrichment for the phosphoinositide 3-kinase/protein kinase B (PI3K/PKB) pathway (Fig. 4C and D). CYTOR is a well-established PI3K/PKB pathway activator, promoting cell survival [46], while CYTOR knockdown reduces PI3K/PKB activity. Furthermore, FOXO3, a primary target inhibited by PIK3/PKB signaling [47], is present in overlapping genes and included in the gene sets for both PI3K/PKB pathway terms. Its up-regulation after CYTOR KD suggests a decrease in PI3K/PKB activity, allowing FOXO3 to escape suppression and become transcriptionally active. FOXO3 has been identified as both an oncogenic gene and a tumor suppressor [48–51]. In gastric cancer, CYTOR is up-regulated and can promote tumor growth through the epidermal growth factor receptor (EGFR)-mediated PI3K/PKB pathway [52]. This mechanism is also identified in glioma [46]. Camptothecin inhibits the progression of nasopharyngeal carcinoma by inhibiting transforming growth factor-beta (TGF-β), PI3K, and PKB expression, but the overexpression of TGF-β mitigates this effect [53]. The observed inverse relationship between CYTOR KD-upregulated genes and camptothecin-downregulated genes suggests that both CYTOR KD and camptothecin may exert a similar suppressive effect on PI3K/PKB signaling. While no direct connection between camptothecin and CYTOR has been previously reported, these findings suggest that in cancers where the PI3K/PKB pathway and CYTOR are highly active, camptothecin may have its most potent anticancer activity.

#### Use case 2: C*rossing the RummaGEO gene sets with the L2S2 gene sets*

**Figure 5.**
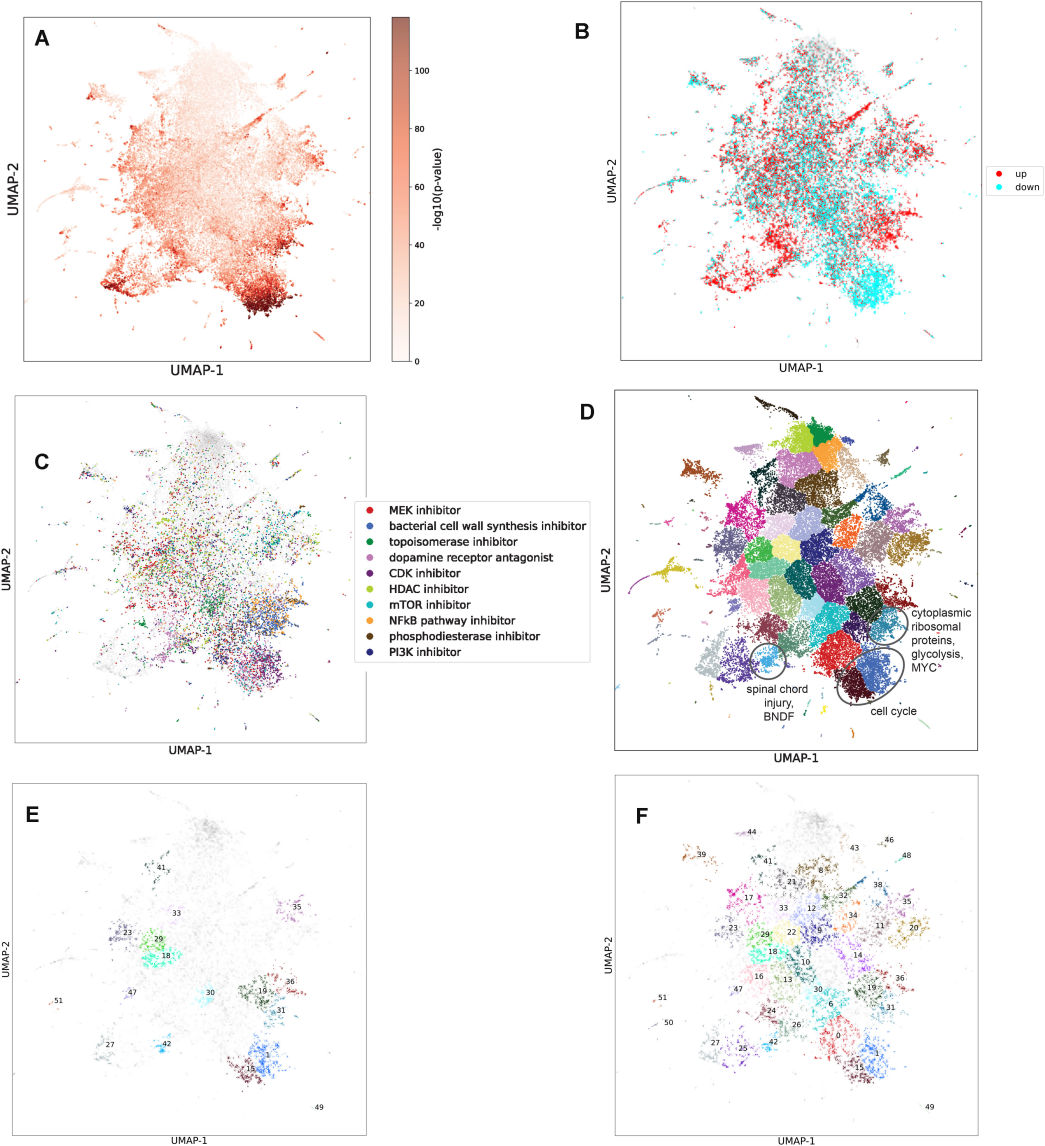
Projection of L2S2 search results on RummaGEO human signatures (*n* = 50 000) UMAP visualization. (**A**) UMAP is annotated by the −log10(*P*-value) of the most significant perturbations from L2S2. (**B**) UMAP is annotated by the directionality of the top ranked perturbation for each RummaGEO gene set. (**C**) UMAP is annotated by the MoA of the top ranked perturbation for each RummaGEO set. (D) UMAP is annotated by the Leiden cluster. (**E**) Leiden clusters annotated by most commonly appearing MoA: C1, CDK inhibitor (0.39); C15, tyrosine kinase inhibitor (0.29); C18, MEK inhibitor (0.16); C19, NF-kB pathway inhibitor, proteasome inhibitor (0.27); C23, MEK inhibitor (0.14); C27, ALK tyrosine kinase receptor inhibitor (0.31); C29, MEK inhibitor (0.18); C30, topoisomerase inhibitor (0.26); C31, NFkB pathway inhibitor, proteasome inhibitor (0.31); C33, HDAC inhibitor (0.15); C35, mTOR inhibitor (0.13); C36, NF-kB pathway inhibitor, proteasome inhibitor (0.36); C41, BCL inhibitor (0.15); C42, dopamine receptor antagonist (0.36); C47, MEK inhibitor (0.24); C49, HSP inhibitor (0.24); C51, sodium channel blocker (0.49). (**F**) Leiden clusters annotated by most commonly appearing disease in the abstracts of each unique study: C0, prostate cancer (0.06); C1, breast cancer (0.07); C6, breast cancer (0.06); C8, Alzheimer’s disease (0.05); C9, prostate cancer (0.07); C10, breast cancer (0.08); C11, acute myeloid leukemia (0.14); C12, Alzheimer’s disease (0.06); C13, breast cancer (0.09); C14, acute myeloid leukemia (0.06); C15, prostate cancer (0.08); C16, breast cancer (0.06); C17, hepatocellular carcinoma (0.07); C18, breast cancer (0.11); C19, breast cancer (0.05); C20, acute myeloid leukemia (0.14); C21, breast cancer (0.06); C22, prostate cancer (0.13); C23, colorectal cancer (0.07); C24, prostate cancer (0.06); C25, sars cov 2 (0.07); C26, sars cov 2 (0.05); C27, sars cov 2 (0.06); C29, breast cancer (0.12); C30, breast cancer (0.07); C31, sars cov 2 (0.07); C32, Alzheimer’s disease (0.1); C33, breast cancer (0.07); C34, acute myeloid leukemia (0.06); C35, acute myeloid leukemia (0.06); C36, sars cov 2 (0.05); C38, Alzheimer’s disease (0.07); C39, Alzheimer’s disease (0.06); C41, prostate cancer (0.08); C42, sars cov 2 (0.08); C43, acute myeloid leukemia (0.07); C44, Alzheimer’s disease (0.06); C46, coronary artery disease (0.09); C47, breast cancer (0.28); C48, microcephaly (0.08); C49, nonsmall cell lung cancer (0.18); C50, breast cancer (0.39); C51, breast cancer (0.51).

To further demonstrate the ability of L2S2 to identify drugs and small molecules that may effectively reverse or mimic these signatures, we sought to systematically cross the L2S2 gene sets with the RummaGEO gene sets. RummaGEO has thousands of differentially expressed genes computed from hundreds of different cell/tissue and disease/phenotype contexts [21]. From RummaGEO, we randomly selected 50 000 gene sets and submitted these gene sets for analysis with L2S2. 87.1% of these queries returned at least one statistically significant enriched perturbation. To assess the distribution of the significant enriched perturbations, the RummaGEO gene sets were visualized as a UMAP where each point represents a gene set. These gene sets are colored by the mean significance of the top perturbations that were returned by L2S2 for each RummaGEO gene set query (Fig. 5A). Several clusters exhibit high concentrations of significant enrichments, while other regions on the UMAP have low or no enrichment for L2S2 perturbations, indicating that these conditions may not be affected by most or all the perturbation profiles in L2S2.

To further explore the functional regions within the UMAP, the RummaGEO gene sets were colored by whether the top enriched perturbation came from an up or a down LINCS L1000 signature (Fig. 5B). The gene sets clearly separate to regions of up and down sets. To further elucidate the shared attributes among the clusters with high enrichment, the MoA of the top-ranked perturbation was visualized on the same UMAP [27] (Fig. 5C). This visualization reveals several drug classes that cluster closely together in clusters of RummaGEO gene sets. For example, dopamine receptor antagonists (light pink) appear to elicit strong enrichment in regions of the darkest clusters in Fig. 5A. At the bottom right of the UMAP, many CDK inhibitor compounds (dark purple) are observed, suggesting a potential link to cell cycle genes. Immediately above this cluster, there are multiple bacterial cell wall synthesis inhibitors and NF-kB pathway inhibitors. This cluster likely contains immune related genes. Another cluster of dopamine receptor antagonists also appears distinctly from other drug classes to the left of the UMAP, while topoisomerase inhibitors spread through a large portion of the middle of the projection. Observing the most commonly overlapping genes in these clusters can help annotate the shared biological mechanisms in each cluster. To explore this, clusters were first automatically identified using the Leiden algorithm [29] (Fig. 5D). Next, consensus genes within each cluster were identified by counting the number of times a gene appears in each set in each cluster. Gene counts exceeding two standard deviations above the mean gene occurrence were considered as the marker genes for each cluster (Supplementary Table S1). Submitting these gene sets for functional analysis with Enrichr [30] revealed each cluster’s MoAs. For instance, the cluster with many dopamine receptor antagonists and MEK inhibitors (cluster 15) is highly enriched for the cell cycle (Reactome Pathways 2024, Cell Cycle, adj. *P*-value 1.21 × e10^-90^) with most genes down-regulated following the given perturbation (Fig. 5B); while the adjacent cluster (cluster 1), enriched for many CDK inhibitors, is also highly enriched for cell cycle genes, but with observed down-regulation (Reactome Pathways 2024, Cell Cycle, adj. *P*-value 2.41 × e10^-96^). This observation aligns with the expected effect of CDK inhibitors, inhibiting the cyclin-dependent kinases [54], which play a crucial role in cell division. MEK inhibitors as well as dopamine receptor antagonists have been also observed to inhibit the cell cycle [55, 56]. The cluster containing mainly dopamine receptor antagonists (cluster 42) is highly enriched for brain-derived neurotrophic factor (BDNF) signaling (BioPlanet 2019, BDNF signaling pathway, adj. *P*-value 5.05 × e10^-48^) and spinal cord injury (WikiPathways 2024 Human, BDNF Spinal Cord Injury WP2431, adj. *P*-value 4.23 × e10^-13^), where the overlaps mainly originate from up-regulated gene sets. BDNF signaling is known to induce the release of dopamine [57], while dopamine receptors have an important role in the spinal cord [58]. The cluster containing many bacterial cell-wall synthesis inhibitors and NFkB pathway inhibitors (cluster 31) contains many cytoplasmic ribosomal proteins (WikiPathways 2024 Human, Cytoplasmic Ribosomal Proteins WP477, adj. *P*-value 2.81 × e10^-122^) as well as enrichment for glycolysis (Elsevier Pathway Collection, Glycolysis, adj. *P*-value 7.08 × e10^-3^). NF-kB activity can modulate the activity of glycolysis and mitochondrial respiration [59]. Overall, the functional enrichment of consensus genes in each cluster seems to align with the predominant known MoA of the enriched drug classes.

To further investigate the predominant diseases and MoAs in the Leiden identified clusters, we assessed the top five enriched compounds for each gene set in each cluster. We then counted the number of times L2S2 enriched drugs with known MoA appear in each cluster. Only clusters where at least 10% of compounds have the same known MoAs were annotated (Fig. 5E). To identify the most prevalent diseases, we extracted disease terms from the abstracts of unique GEO series and retained clusters where at least 5% of the annotations were consistent (Fig. 5F). Consistent with the MoA of the top perturbation, the consensus MoA in cluster 1 is CDK inhibitors (39%). In cluster 15, a large portion of the highly enriched compounds are tyrosine kinase inhibitors (29%) which aligns with the downregulation of cell cycle. Cluster 1 has many gene sets related to breast cancer (7%), while cluster 15 has a high occurrence of gene sets related to prostate cancer (8%). CDK inhibitors have been successfully utilized in breast cancer [60]. Additionally, tyrosine kinase inhibitors have been studied extensively for the treatment of prostate cancer [61, 62], though none have been FDA-approved due to unsuccessful clinical trials. Both MEK inhibitors and dopamine receptor antagonists have also been identified as potential therapeutics in the treatment of prostate cancer [63, 64]. Additionally, cluster 42 has a high prevalence of SARS-CoV-2 related gene sets (8%) which has been shown to disrupt serum BDNF in severe cases [65], which is also enriched in this cluster. Dopamine receptor antagonists were the predominant MoA in cluster 42 (36%) and have been proposed as therapeutics for treating COVID-19 [66] due to the virus’ influence on dopaminergic signaling to reduce immune response. Applying the identified drug classes to the most prevalent disease annotations in each cluster highlights the potential for drug repurposing across diverse diseases and other conditions.

## Discussion and conclusions

By serving 1.678 million gene sets created from the experimental results of profiling 248 cell lines in response to 33 621 small molecules and 7508 single-gene knockouts, L2S2 provides a next-generation LINCS L1000 search engine that may enable drug repurposing, target discovery, and the discovery of novel MoA for drug, small molecules, and single gene knockouts. Beyond ranking individual signatures, L2S2 enables the identification of compounds and other perturbations that influence the mRNA expression of a query gene set across diverse cellular contexts, concentrations, and time points by performing consensus perturbation enrichment analysis. For consensus enrichment analysis, we developed and benchmarked three strategies. We show that the Top-N consensus signature search method most effectively recovers expected compounds when querying L2S2 with manually curated drug-response gene expression profiles from published microarray studies. Additionally, we demonstrate the utility of L2S2 in identifying compounds that may mimic the knockdown of the lncRNA CYTOR. By indirectly targeting CYTOR with the anticancer drug camptothecin, we expect to improve patient outcomes for cancers that highly express CYTOR and display high PI3K/PKB pathway activity. L2S2 APIs speed and scale enable its application for global analyses as illustrated by our application to analyze thousands of human gene sets served by RummaGEO [21]. This analysis revealed connections between drug classes, specific compounds and drugs, diseases, and tissues. The global visualization of such relationships can be used to obtain a global view of pharmacology to discover novel targets, drugs, drug MoAs, and mechanisms of disease.

Given the broad coverage of gene sets and perturbations, L2S2 can be systematically applied to identify mimickers and reversers to many more disease signatures. Specifically, we plan to apply L2S2 to tumor-specific gene expression signatures derived from patient cohorts from Clinical Proteomic Tumor Analysis Consortium (CPTAC), apply L2S2 to predict potential side effects for preclinical drugs, and examine the MoAs of preclinical uncharacterized compounds. The L2S2 transcriptomics-based approach to drug and target discovery complements traditional drug development pipelines which are target focused. L2S2 can be used to rapidly advance compounds for rare diseases with limited therapeutic options, emerging infectious diseases, and complex conditions such as neurodegenerative or autoimmune disorders, as well as suggest personalized treatments.

While the L2S2 web server application provides opportunities for promising applications, some limitations should be considered. For example, the L1000 assay only directly measures ∼1000 genes, while the expression of ∼11 000 additional genes is inferred via an extrapolation algorithm. This means less accuracy compared with methods such as bulk RNA-seq that directly measure the expression of all genes. In addition, the 1.678 million gene sets in L2S2 comprises of only ∼12 000 protein-coding genes. This is only approximately half of the known human coding genes. Such lack of coverage can impact enrichment analysis accuracy. Alternative methods to increase such coverage have been proposed [67]. Additionally, the number of signatures per perturbation varies significantly. This leads to over-representation of certain perturbations in enrichment results. This issue is somewhat mitigated by the consensus rankings. However, compounds with many signatures still have some statistical advantage to rank higher compared with less frequently profiled compounds.

There are various options to compare and represent signatures: gene set versus gene set; gene set versus a vector of ranked genes; and a vector of ranked genes versus another vector of ranked genes. L2S2 utilizes the gene set versus gene set enrichment-based approach. However, other methods should outperform set vs. set methods. This is because vectors of ranked genes include more information extracted from the original data when comparing gene expression profiles. The disadvantage of correlation-based methods is that they require more complex algorithms that might be slower to implement. Furthermore, inconsistency across assays, studies, and experiments requires normalization strategies. In contrast, the gene set enrichment approach, used by L2S2, offers a more standardized and interpretable framework, enabling robust comparisons across diverse datasets at a quicker speed.

In the most recent release of the L1000 data, and within the L2S2 search engine, there are signatures of 7508 single-gene CRISPR knockouts in at least 10 diverse cell lines. Such a rich dataset is still under-utilized. It has the potential to match knockouts to drugs to deorphanize drugs, and match knockouts to disease signatures to discover novel targets and pathways. The L2S2 fast API should be able to enable such novel analyses. Overall, L2S2 enables the biomedical research community to easily query, search, and analyze the most recently published LINCS L1000 dataset to facilitate hypothesis generation for many future studies.

## Supplementary Material

gkaf373_Supplemental_Files

## Data Availability

The L2S2 search engine is available from https://l2s2.maayanlab.cloud/. The LINCS L1000 processed signatures are available on the download page at https://l2s2.maayanlab.cloud/download. The L2S2 source code is available from https://github.com/MaayanLab/L2S2. A snapshot of the code has been deposited at https://doi.org/10.5281/zenodo.15256496.

## Notes

Present address: Department of Pharmacological Sciences, Department of Artificial Intelligence and Human Health, Mount Sinai Center for Bioinformatics, One Gustave L. Levy Place, Box 1603, Icahn School of Medicine at Mount Sinai, New York, NY 10029, United States
