# Supplementary material for: L2S2: chemical perturbation and CRISPR KO LINCS L1000 signature search engine
Source: Nucleic Acids Res. 2025 May 1;53(W1):W338–50. doi: 10.1093/nar/gkaf373 (PMC12230732; doi:10.1093/nar/gkaf373)
Supplement: gkaf373_Supplemental_Files [file gkaf373_supplemental_files.zip › Supplementary Figures.pdf]

Figure S1. Methodology for **A.** Up-and-down gene set search significance computation. N-User-Signature is equal to the length of the up-user gene set added to the length of the down-user gene set. N-L100-Signature equals the length of the up-gene set added to the length of the down gene set (n=500) and **B.** Consensus perturbation p-value computation for up and down/mimicker reverser compounds.

Figure S2. Benchmarking the Top-N (500, 1000, 5000, 10,000, 20,000, 40,000, 50,0000, 75,0000) consensus perturbation rankings by submitting up- and down-regulated gene sets from curated drug perturbation studies from GEN3VA compared to mean characteristic direction gene sets from SigCom LINCS; A. ROC curves of ranked perturbations for dexamethasone (n=86) up-regulated genes; B. ROC curves of ranked perturbations for dexamethasone (n=86) down-regulated genes; C. ROC curves of ranked perturbations for thiazolidinedione (n=94) up-regulated genes; D. ROC curves of ranked perturbations for thiazolidinedione (n=94) down-regulated genes; E. ROC curves of ranked perturbations for tamoxifen (n=66) up-regulated genes; F. ROC curves of ranked perturbations for tamoxifen (n=66) down-regulated genes.

Table S1. Leiden clusters assigned to 50,000 RummaGEO signatures, consensus genes per cluster, and links to enrichment results from Enrichr.

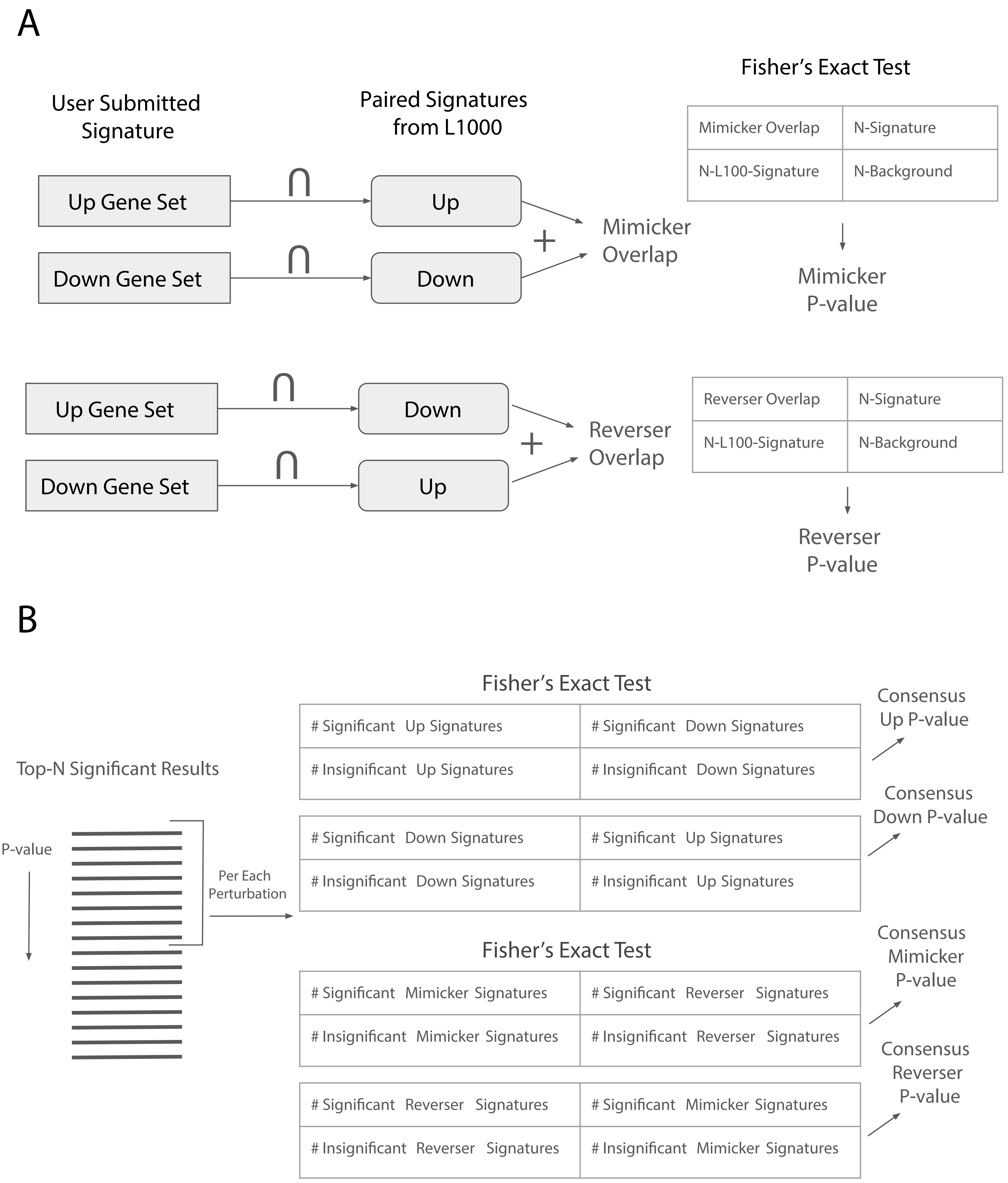
A
User Submitted Signature
Paired Signatures from L1000
Fisher's Exact Test
Up Gene Set
Down Gene Set
∩
Up
Down
+
Mimicker Overlap
Mimicker Overlap
N-Signature
N-L100-Signature
N-Background
Mimicker P-value
Up Gene Set
Down Gene Set
Down
Up
Reverser Overlap
Reverser Overlap
N-Signature
N-L100-Signature
N-Background
Reverser P-value
B
Fisher's Exact Test
Significant Up Signatures
Significant Down Signatures
Insignificant Up Signatures
Insignificant Down Signatures
Consensus Up P-value
Top-N Significant Results
Significant Down Signatures
Significant Up Signatures
Insignificant Down Signatures
Insignificant Up Signatures
Consensus Down P-value
P-value
Per Each Perturbation
Fisher's Exact Test
Significant Mimicker Signatures
Significant Reverser Signatures
Insignificant Mimicker Signatures
Insignificant Reverser Signatures
Consensus Mimicker P-value
Significant Reverser Signatures
Significant Mimicker Signatures
Insignificant Reverser Signatures
Insignificant Mimicker Signatures
Consensus Reverser P-value

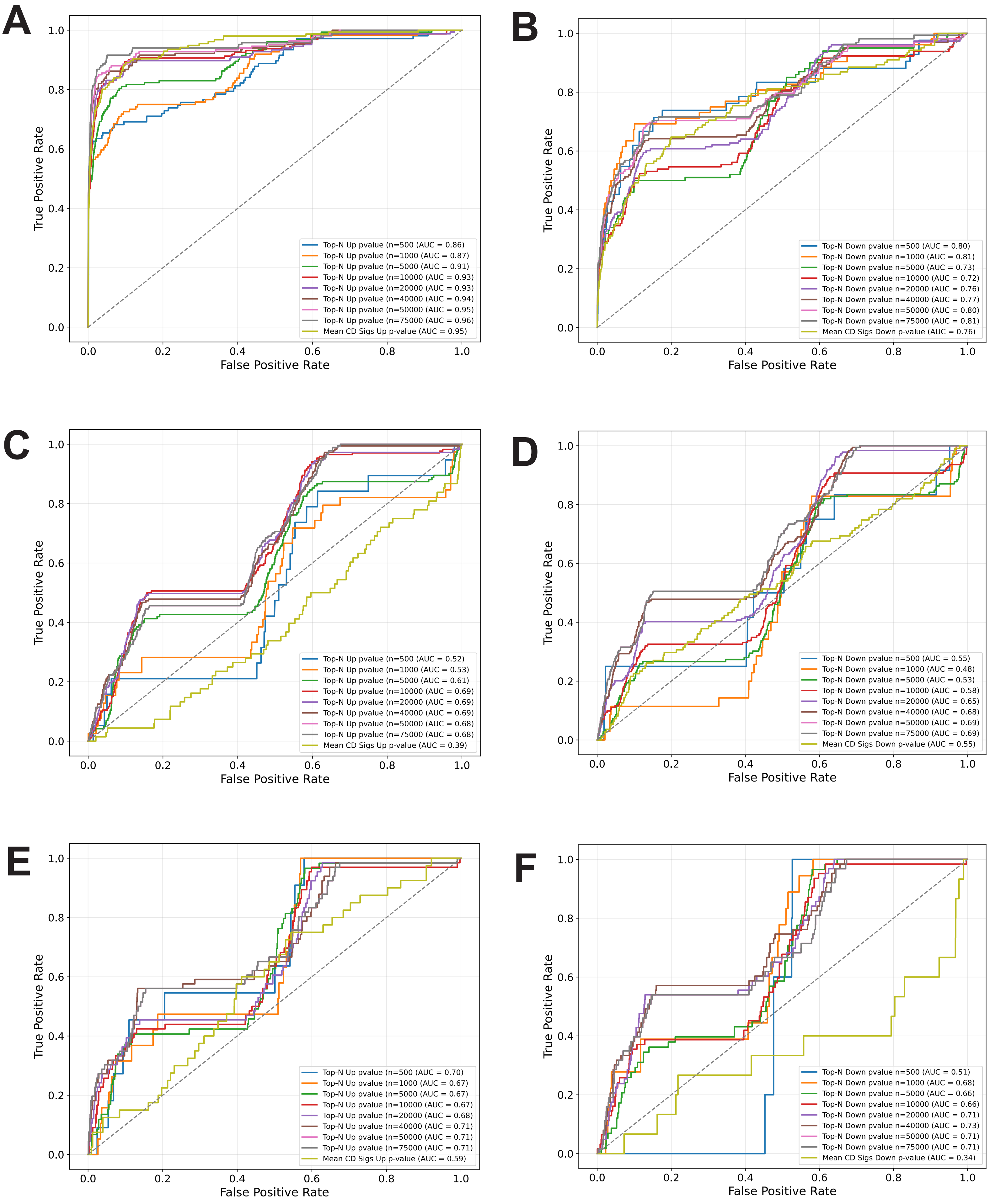
A
True Positive Rate
False Positive Rate
Top-N Up pvalue (n=500 (AUC = 0.86)
Top-N Up pvalue (n=1000 (AUC = 0.87)
Top-N Up pvalue (n=5000 (AUC = 0.91)
Top-N Up pvalue (n=10000 (AUC = 0.93)
Top-N Up pvalue (n=20000 (AUC = 0.93)
Top-N Up pvalue (n=40000 (AUC = 0.94)
Top-N Up pvalue (n=50000 (AUC = 0.95)
Top-N Up pvalue (n=75000 (AUC = 0.96)
Mean CD Sigs Up p-value (AUC = 0.95)
B
True Positive Rate
False Positive Rate
Top-N Down pvalue n=500 (AUC = 0.80)
Top-N Down pvalue n=1000 (AUC = 0.81)
Top-N Down pvalue n=5000 (AUC = 0.73)
Top-N Down pvalue n=10000 (AUC = 0.72)
Top-N Down pvalue n=20000 (AUC = 0.76)
Top-N Down pvalue n=40000 (AUC = 0.77)
Top-N Down pvalue n=50000 (AUC = 0.80)
Top-N Down pvalue n=75000 (AUC = 0.81)
Mean CD Sigs Down p-value (AUC = 0.76)
C
True Positive Rate
False Positive Rate
Top-N Up pvalue (n=500 (AUC = 0.52)
Top-N Up pvalue (n=1000 (AUC = 0.53)
Top-N Up pvalue (n=5000 (AUC = 0.61)
Top-N Up pvalue (n=10000 (AUC = 0.69)
Top-N Up pvalue (n=20000 (AUC = 0.69)
Top-N Up pvalue (n=40000 (AUC = 0.69)
Top-N Up pvalue (n=50000 (AUC = 0.68)
Top-N Up pvalue (n=75000 (AUC = 0.68)
Mean CD Sigs Up p-value (AUC = 0.39)
D
True Positive Rate
False Positive Rate
Top-N Down pvalue n=500 (AUC = 0.55)
Top-N Down pvalue n=1000 (AUC = 0.48)
Top-N Down pvalue n=5000 (AUC = 0.53)
Top-N Down pvalue n=10000 (AUC = 0.58)
Top-N Down pvalue n=20000 (AUC = 0.65)
Top-N Down pvalue n=40000 (AUC = 0.68)
Top-N Down pvalue n=50000 (AUC = 0.69)
Top-N Down pvalue n=75000 (AUC = 0.69)
Mean CD Sigs Down p-value (AUC = 0.55)
E
True Positive Rate
False Positive Rate
Top-N Up pvalue (n=500 (AUC = 0.70)
Top-N Up pvalue (n=1000 (AUC = 0.67)
Top-N Up pvalue (n=5000 (AUC = 0.67)
Top-N Up pvalue (n=10000 (AUC = 0.67)
Top-N Up pvalue (n=20000 (AUC = 0.68)
Top-N Up pvalue (n=40000 (AUC = 0.71)
Top-N Up pvalue (n=50000 (AUC = 0.71)
Top-N Up pvalue (n=75000 (AUC = 0.71)
Mean CD Sigs Up p-value (AUC = 0.59)
F
True Positive Rate
False Positive Rate
Top-N Down pvalue n=500 (AUC = 0.51)
Top-N Down pvalue n=1000 (AUC = 0.68)
Top-N Down pvalue n=5000 (AUC = 0.66)
Top-N Down pvalue n=10000 (AUC = 0.66)
Top-N Down pvalue n=20000 (AUC = 0.71)
Top-N Down pvalue n=40000 (AUC = 0.73)
Top-N Down pvalue n=50000 (AUC = 0.71)
Top-N Down pvalue n=75000 (AUC = 0.71)
Mean CD Sigs Down p-value (AUC = 0.34)
